# Therapeutic Strategies After Immunotherapy Failure in Advanced Non-Small Cell Lung Cancer: Mechanisms of Resistance and Emerging Treatment Approaches

**DOI:** 10.3390/cancers18213523

**Published:** 2026-11-02

**Authors:** Masatoshi Kanayama, Masaru Takenaka, Hidetaka Uramoto

**Affiliations:** Second Department of Surgery, University of Occupational and Environmental Health, 1-1 Iseigaoka, Yahatanishi-ku, Kitakyushu 807-8555, Japan; m-takenaka@med.uoeh-u.ac.jp (M.T.); uramoto@med.uoeh-u.ac.jp (H.U.)

**Keywords:** non-small cell lung cancer, immune checkpoint inhibitors, immunotherapy failure, treatment resistance, chemotherapy, local therapy, salvage surgery, immune checkpoint inhibitor rechallenge, biomarkers

## Abstract

Immune checkpoint inhibitors (ICIs) have transformed the treatment landscape of advanced non-small cell lung cancer (NSCLC), but disease progression during or after immunotherapy remains a major therapeutic challenge. ICI failure is biologically and clinically heterogeneous and may manifest as primary resistance, acquired resistance, oligoprogressive disease, hyperprogressive disease, or disease recurrence following treatment discontinuation. These distinct patterns may require different therapeutic approaches. In this review, we summarize the current evidence for treatment strategies after immunotherapy failure in advanced NSCLC. Cytotoxic chemotherapy remains a key subsequent-line treatment, though evidence supporting enhanced chemotherapy sensitivity after prior ICI exposure is largely derived from retrospective or non-randomized studies. Docetaxel plus ramucirumab is an established treatment option, but its specific efficacy after ICI failure has not been confirmed in randomized trials. Local therapies, including stereotactic body radiotherapy and salvage surgery, may provide meaningful local control in carefully selected patients with oligoprogressive or otherwise localized disease, though prospective evidence remains limited. ICI rechallenge generally has limited activity after progression during active ICI treatment, whereas re-exposure may be considered in selected patients after planned treatment completion or discontinuation because of immune-related adverse events. Biomarker development, including tissue biomarkers, circulating tumor DNA, circulating tumor cells, and emerging tumor–immune profiling approaches, may facilitate more individualized treatment selection. Overall, management after immunotherapy failure should be guided by the pattern and tempo of disease progression, prior treatment history, and the feasibility of systemic and local treatment. Further prospective studies are needed to establish effective treatment strategies and validated biomarkers for patient selection.

## Simple Summary

Although immune checkpoint inhibitors (ICIs) have substantially improved outcomes for patients with advanced non-small cell lung cancer (NSCLC), disease progression during or after ICI therapy remains a major clinical challenge. ICI failure is heterogeneous and includes primary resistance, acquired resistance, oligoprogressive disease, hyperprogressive disease, and disease recurrence after treatment discontinuation. Subsequent treatment should therefore be individualized according to the pattern and extent of disease progression, previous treatment, and patient characteristics. Cytotoxic chemotherapy remains an important treatment option, while local therapies such as radiotherapy and salvage surgery may provide disease control in selected patients. ICI rechallenge may also be considered in selected clinical settings, particularly after planned treatment completion or discontinuation for reasons other than disease progression. Emerging biomarkers may further guide treatment selection. This review summarizes current evidence and discusses treatment strategies and future directions for patients with advanced NSCLC after immunotherapy failure.

## 1. Introduction

Over the past decade, immune checkpoint inhibitors (ICIs) targeting programmed cell death protein 1 (PD-1), programmed death ligand 1 (PD-L1), and cytotoxic T-lymphocyte-associated protein 4 (CTLA-4) have transformed the therapeutic landscape of non-small cell lung cancer (NSCLC), demonstrating substantial clinical benefit across multiple treatment settings, including advanced disease, unresectable stage III disease following concurrent chemoradiotherapy, and resectable disease in the perioperative setting. In advanced NSCLC, landmark clinical trials such as KEYNOTE-024, KEYNOTE-189, KEYNOTE-407, IMpower150, CheckMate 227, and CheckMate 9LA established ICI-based regimens as standard treatment options across diverse patient populations [1,2,3,4,5,6]. More recently, the therapeutic role of ICIs has expanded into earlier-stage disease, with neoadjuvant and perioperative trials including CheckMate 816, KEYNOTE-671, AEGEAN, and CheckMate 77T demonstrating the clinical benefit of incorporating ICIs into multimodality treatment strategies [7,8,9,10].

The optimal management of patients with disease progression during or after immunotherapy remains challenging because ICI resistance is biologically heterogeneous and may involve diverse tumor-intrinsic and immune-mediated mechanisms [11,12,13,14]. Consequently, evidence guiding treatment selection after ICI failure remains limited, with much of the available evidence derived from retrospective studies, real-world data, and expert consensus rather than prospective clinical trials [15,16].

Disease progression during or after ICI therapy encompasses several clinically distinct patterns, including primary resistance, acquired resistance, oligoprogression, hyperprogressive disease, and progression after treatment discontinuation [12,17,18,19]. Immunotherapy failure should therefore not be regarded as a single clinical entity. These distinct patterns may require different therapeutic approaches, underscoring the importance of individualized treatment strategies that integrate systemic and local therapies [17].

Accordingly, several key clinical questions remain unresolved: What is the optimal role of systemic therapy after ICI failure? Which patients may benefit from ICI rechallenge or local ablative therapy for oligoprogressive disease [16,17]? Can salvage surgery provide durable disease control in highly selected patients [20]? Finally, can emerging biomarkers—including circulating tumor DNA (ctDNA), circulating tumor cells (CTCs), tumor immune microenvironment profiling, and dynamic immune biomarkers—facilitate personalized treatment selection after immunotherapy failure [21]?

In this review, we comprehensively summarize current evidence regarding therapeutic strategies after immunotherapy failure in advanced NSCLC. We first discuss mechanisms and patterns of resistance, systemic treatment options including chemotherapy and ICI rechallenge, local treatment approaches such as radiotherapy and salvage surgery, and emerging biomarkers that may facilitate precision treatment selection. By integrating evidence from clinical trials, translational research, and real-world studies, this review provides a practical framework for optimizing post-immunotherapy management in contemporary thoracic oncology.

## 2. Patterns of Immunotherapy Failure

Immunotherapy failure encompasses several clinically and biologically distinct patterns. The Society for Immunotherapy of Cancer (SITC) has proposed standardized consensus definitions of immunotherapy resistance, providing a framework for classifying resistance according to the timing of progression, prior clinical benefit, and treatment context [12,22,23,24]. Initially developed for ICI monotherapy, these definitions have subsequently been expanded to include combination regimens incorporating chemotherapy, dual immune checkpoint blockade, and molecularly targeted therapies [22,23,24].

Recognizing the pattern of immunotherapy failure is increasingly important because subsequent treatment decisions are largely influenced by the underlying resistance phenotype. Patients with primary resistance generally require an early change in systemic therapy, whereas patients with acquired resistance may be considered for treatment strategies tailored to the extent and tempo of disease progression. In contrast, patients with oligoprogressive disease may remain candidates for local ablative therapy with continuation of systemic treatment in selected cases. Similarly, disease progression following treatment discontinuation, including discontinuation because of immune-related adverse events (irAEs), represents a distinct clinical scenario requiring individualized treatment consideration.

This section summarizes the major patterns of immunotherapy failure in NSCLC, including primary resistance, acquired resistance, oligoprogressive disease, hyperprogressive disease, and disease progression following treatment discontinuation, with emphasis on their underlying biology and clinical implications.

### 2.1. Primary Resistance

Primary resistance refers to the absence of clinically meaningful benefit despite adequate exposure to ICIs. According to the SITC consensus definitions for single-agent PD-1/PD-L1 blockade, primary resistance is generally defined as disease progression within 6 months of treatment initiation after at least 6 weeks of therapy, or stable disease lasting less than 6 months. When disease progression is observed, confirmatory imaging is generally recommended at least 4 weeks after the initial evidence of progression to confirm progressive disease and account for the possibility of delayed responses or pseudoprogression, provided that the patient remains clinically stable. In patients with rapid clinical deterioration attributable to disease progression, treatment modification may be appropriate without waiting for confirmatory imaging [12].

Primary resistance remains a major clinical challenge in patients with advanced NSCLC treated with ICI-based therapy. Its reported frequency varies according to PD-L1 expression, treatment regimen, histologic subtype, disease stage, and tumor genomic characteristics. The biological basis of primary resistance is multifactorial, reflecting complex interactions among tumor-intrinsic factors, the tumor microenvironment, and host immune responses [25,26].

Tumor-intrinsic mechanisms include impaired antigen presentation, defective interferon-γ signaling, and low tumor immunogenicity. Defects in HLA class I molecules or β2-microglobulin (B2M) can impair tumor antigen presentation and recognition by cytotoxic CD8+ T cells. In addition, inadequate dendritic-cell priming, impaired T-cell trafficking or infiltration, and the presence of immune-desert or immune-excluded tumor microenvironments may limit effective antitumor immune responses. Immunosuppressive populations, including myeloid-derived suppressor cells and regulatory T cells, as well as alternative inhibitory immune pathways, may further contribute to primary resistance [25,26].

Tumor genomic alterations have also been implicated in primary resistance to PD-1/PD-L1 blockade in NSCLC. In particular, STK11 alterations have been associated with reduced sensitivity to PD-1/PD-L1 blockade and identified as an important genomic determinant of primary resistance in KRAS-mutant lung adenocarcinoma [27]. Alterations in KEAP1/NFE2L2 have also been associated with poorer outcomes following immune therapy in KRAS-mutant NSCLC, though their predictive role remains less clearly established [28].

NSCLC harboring activating EGFR mutations or ALK rearrangements generally exhibits limited sensitivity to ICI monotherapy. Several biological factors may contribute to this phenomenon, including relatively low tumor mutational burden, reduced neoantigen availability, and a less inflamed tumor microenvironment characterized by limited CD8-positive T-cell infiltration and impaired antitumor immune activation. In addition, oncogenic driver signaling may promote immunosuppressive cellular and cytokine networks that facilitate immune evasion [29,30].

From a clinical perspective, patients with confirmed primary resistance to ICI monotherapy are unlikely to derive meaningful benefit from continued PD-1/PD-L1 blockade alone. Subsequent treatment should therefore generally be directed toward an appropriate alternative systemic strategy based on prior therapy, histologic and molecular characteristics, performance status, and patient preferences. Cytotoxic chemotherapy remains an important treatment option in patients without actionable molecular alterations, while clinical trial enrollment should be considered whenever available [31].

Although these principles were initially established for single-agent PD-1/PD-L1 blockade, primary resistance also occurs with ICI-based combination regimens, for which SITC has proposed separate resistance definitions according to the treatment context [22,23,24]. Importantly, although the biological mechanisms described above may be relevant to ICI resistance across different treatment settings, the extent to which each mechanism contributes to resistance to combination regimens remains incompletely understood. In patients receiving ICI–chemotherapy combinations, disease progression is generally evaluated in the context of the combination regimen as a whole, and the clinical course alone cannot establish the relative contribution of the ICI and chemotherapy components [22].

### 2.2. Acquired Resistance

Acquired resistance develops after an initial objective response or durable disease stabilization and is characterized by disease progression following a period of clinically meaningful benefit from ICIs. For single-agent PD-1/PD-L1 blockade, the SITC consensus definitions generally classify acquired resistance as disease progression after at least 6 months of clinical benefit, provided that adequate treatment exposure has been achieved [12].

Unlike primary resistance, acquired resistance is thought to arise under sustained immune selection pressure exerted by effective immunotherapy. During prolonged treatment, tumor evolution may favor the emergence or expansion of resistant subclones capable of escaping immune surveillance despite an initially effective antitumor immune response. This process reflects the dynamic interaction between tumor cells and the host immune system and may involve both tumor-intrinsic genetic alterations and remodeling of the tumor immune microenvironment [25,32].

Several molecular mechanisms have been implicated in acquired resistance: immunoediting and the loss of immunogenic neoantigens may reduce tumor recognition by previously activated T cells [25]; impaired antigen processing and presentation caused by alterations in HLA class I molecules or B2M can provide another mechanism of immune escape [14,25]; disruption of interferon-γ signaling through alterations in JAK1 or JAK2 may further compromise tumor-cell responses to antitumor immune activity [25,32]; and progressive T-cell dysfunction and remodeling of the tumor immune microenvironment may also facilitate immune escape during treatment [25,32].

Longitudinal studies in NSCLC provide evidence supporting the dynamic nature of acquired resistance. In an analysis of matched pretreatment and post-ICI tumor samples from 82 patients with NSCLC who developed acquired resistance, the treatment regimens included PD-(L)1 inhibitor monotherapy (56.1%), combined PD-1 and CTLA-4 blockade (6.1%), and chemoimmunotherapy (37.8%). Acquired alterations in STK11, B2M, APC, MTOR, KEAP1, and JAK1/2 were identified in a subset of patients. Post-treatment samples also showed reduced intratumoral CD3+ and CD8+ T-cell infiltration and decreased HLA class I expression. These findings support the potential contribution of both tumor evolution and changes in the immune microenvironment to acquired resistance [32]. However, whether the relative contributions of these mechanisms differ across ICI treatment regimens remains incompletely understood.

### 2.3. Oligoprogressive Disease

Oligoprogressive disease (OPD) represents a distinct clinical pattern of treatment failure in which disease progression is confined to a limited number of metastatic lesions, while the remaining tumor burden remains controlled by ongoing systemic therapy [33,34].

The biological basis of oligoprogression is thought to reflect spatial tumor heterogeneity. Under the selective pressure of systemic therapy, resistant tumor subclones may emerge within individual lesions, whereas other metastatic sites may remain sensitive to ongoing treatment [11,25]. This pattern provides a biological rationale for local eradication of resistant lesions while maintaining systemic therapy that continues to control the remaining disease [33,34].

Local ablative therapy, including stereotactic body radiation therapy (SBRT) and, in highly selected cases, surgical resection, may therefore be considered for patients with oligoprogressive NSCLC in whom the progressing lesions are amenable to definitive local treatment while the remaining disease remains controlled by systemic therapy.

### 2.4. Hyperprogressive Disease

Hyperprogressive disease (HPD) is a distinct pattern of disease progression characterized by a paradoxical acceleration of tumor growth shortly after the initiation of ICI therapy. However, no universally accepted diagnostic criteria have been established, and reported incidences vary substantially according to the definition used. Systematic reviews have reported HPD rates ranging from approximately 6% to 43% across studies, highlighting the substantial heterogeneity in its assessment [35,36].

Several clinical and biological factors have been associated with HPD, though none has been established as a validated predictive biomarker. In NSCLC, a high metastatic burden, including involvement of more than two metastatic sites, has been associated with an increased risk of HPD [18,35]. Tumor genomic alterations, particularly MDM2/MDM4 amplification and EGFR alterations, have also been implicated in hyperprogression [37]. In addition, alterations in the tumor immune microenvironment may contribute to HPD. In a study of patients with NSCLC, HPD was associated with increased infiltration of myeloid cells and the presence of CD163+CD33+PD-L1+ macrophages, suggesting that Fc receptor-mediated interactions between anti-PD-1 antibodies and immunosuppressive macrophages may contribute to accelerated tumor growth [38].

Because HPD is associated with rapid clinical deterioration and poor clinical outcomes, early recognition is critical. In a multicenter cohort of patients with advanced NSCLC treated with PD-1/PD-L1 inhibitors, 93.6% received ICI monotherapy, and HPD was identified in 13.8% of patients, associated with a median overall survival of only 3.4 months (95% CI, 2.8–7.5 months) [18]. When rapid clinical or radiographic progression occurs shortly after initiation of ICI therapy, HPD should therefore be considered, particularly in patients with a high tumor burden or rapid deterioration. Once HPD is suspected or confirmed, continuation of ineffective ICI therapy should generally be avoided, and an alternative treatment strategy should be considered promptly.

### 2.5. Progression After Treatment Discontinuation

IrAEs may necessitate temporary interruption or permanent discontinuation of ICI therapy. Nevertheless, antitumor activity may persist after treatment discontinuation, and some patients maintain prolonged disease control despite cessation of therapy. A pooled analysis of prospective cohort studies evaluated 835 patients with advanced NSCLC who discontinued ICI therapy. Patients who discontinued treatment after achieving a durable tumor response had particularly favorable post-discontinuation outcomes [19].

Disease progression after ICI discontinuation represents a distinct clinical scenario from progression during active treatment. In selected patients who previously achieved clinical benefit, ICI resumption or rechallenge may be considered depending on the reason for discontinuation, prior treatment response, and the risk of recurrent toxicity [39,40]. However, this approach requires careful patient selection because recurrent or new irAEs may occur after treatment resumption. The decision should balance the potential for renewed antitumor activity against the risk of recurrent or severe immune-related toxicity.

### 2.6. Clinical Implications

**Figure 1:**
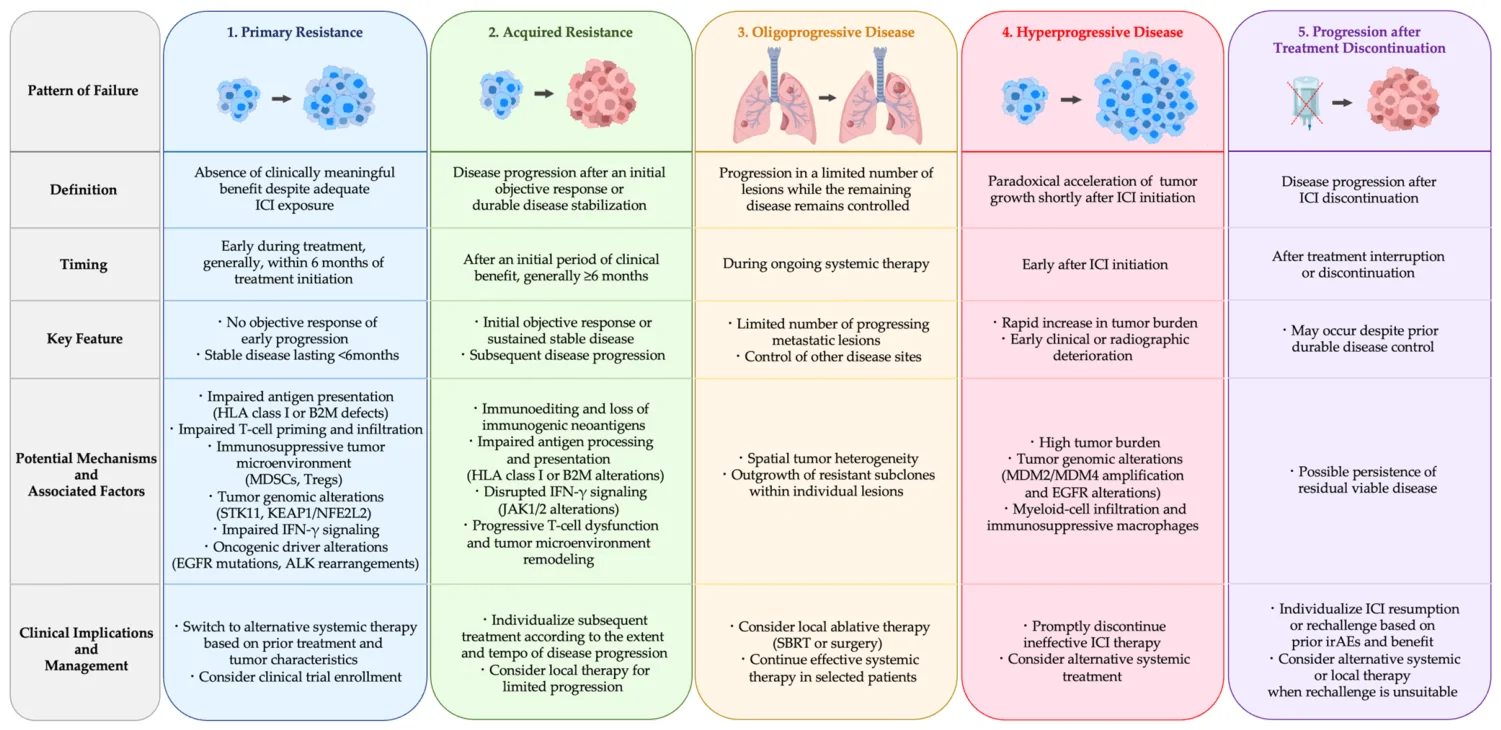
Major patterns of immunotherapy failure in lung cancer and corresponding management strategies. Abbreviations: ICI, immune checkpoint inhibitor; HLA, human leukocyte antigen; B2M, beta-2-microglobulin; MDSCs, myeloid-derived suppressor cells; Tregs, regulatory T cells; STK11, serine/threonine kinase 11; KEAP1, Kelch-like ECH-associated protein 1; NFE2L2, nuclear factor erythroid 2 like 2; IFN-γ, interferon-gamma; EGFR, epidermal growth factor receptor; ALK, anaplastic lymphoma kinase; JAK1/2, Janus kinase 1/2; SBRT, stereotactic body radiation therapy; MDM2, mouse double minute 2 homolog; MDM4, mouse double minute 4 homolog; irAEs, immune-related adverse events.

Accurate characterization of immunotherapy failure patterns should form the basis of individualized post-ICI management (Figure 1). Patients with primary resistance generally require a change in systemic therapy, whereas selected patients with acquired resistance or oligoprogressive disease may be candidates for continued immunotherapy, local ablative treatment, or a combination of systemic and local approaches [12,17,33,34]. In patients who discontinue ICI therapy because of irAEs, subsequent treatment decisions require careful assessment of the prior antitumor benefit, the severity and type of irAE, and the potential risk of recurrent immune-related toxicity [16,39,40].

## 3. Chemotherapy After Immunotherapy Failure

### 3.1. Clinical Evidence

Despite remarkable advances in immunotherapy, cytotoxic chemotherapy remains an important systemic treatment option for patients who experience disease progression following ICI therapy. For patients with advanced NSCLC without actionable driver alterations who develop progression during or after first-line ICI-based treatment, subsequent therapy is generally selected according to prior chemotherapy exposure, histologic subtype, performance status, and other clinical factors. Current international guidelines recommend standard cytotoxic chemotherapy options for patients who progress during or after first-line ICI-based therapy [31,41].

Accumulating retrospective evidence suggests that chemotherapy administered after ICI exposure retains clinically meaningful antitumor activity and may exhibit enhanced activity in some patients. In a retrospective study of 73 patients with NSCLC, Park et al. reported an objective response rate (ORR) of 53.4% with salvage chemotherapy administered after PD-1/PD-L1 inhibitor therapy, compared with 34.9% with the last chemotherapy administered before immunotherapy (*p* = 0.03) [42]. The difference was particularly pronounced among patients receiving platinum-based doublet chemotherapy, for whom the ORR was 66.7% after immunotherapy compared with 39.5% before immunotherapy. Another retrospective study by Schvartsman et al. reported an ORR of 39.3% with single-agent chemotherapy administered after prior PD-1 inhibitor therapy [43].

Several other retrospective studies have reported similar findings. In a Japanese multicenter analysis, subsequent chemotherapy after nivolumab was associated with an ORR of 28.6% and a median PFS of 5.26 months when administered immediately after nivolumab, compared with an ORR of 16.4% and a median PFS of 2.74 months among patients who received comparable chemotherapy without prior ICI exposure [44]. In the WJOG10217L study, a propensity score-weighted analysis of 1439 patients with advanced or recurrent NSCLC, chemotherapy administered after PD-1 inhibitor therapy was associated with a higher response rate than chemotherapy without prior PD-1 inhibitor exposure (18.9% vs. 11.0%). However, no significant differences were observed in PFS (2.8 vs. 2.7 months; *p* = 0.55) or overall survival (OS; 9.2 vs. 10.4 months; *p* = 0.63) [45]. Collectively, these findings indicate that chemotherapy remains active after ICI exposure, though whether prior ICI treatment enhances the efficacy of subsequent chemotherapy remains uncertain.

The biological basis of this potential enhancement of chemotherapy sensitivity remains uncertain. Proposed mechanisms include chemotherapy-induced immunogenic cell death and modulation of the tumor immune microenvironment, which may enhance antitumor immune activity [46]. However, these mechanisms remain largely hypothetical in the specific setting of chemotherapy administered after ICI exposure, and the available clinical evidence cannot establish a causal relationship between prior ICI therapy and enhanced chemotherapy sensitivity.

Importantly, higher response rates do not necessarily translate into more durable disease control or improved survival. In the retrospective study by Park et al., median PFS did not differ significantly between chemotherapy administered after PD-1/PD-L1 inhibitor therapy and the last chemotherapy administered before immunotherapy (4.2 vs. 4.7 months; *p* = 0.84) [42]. Together with the WJOG10217L findings, these results underscore the importance of distinguishing tumor response from longer-term clinical outcomes. Interpretation is further limited by heterogeneity in prior ICI duration, treatment line, type of ICI, chemotherapy regimen, PD-L1 expression, molecular characteristics, and patient selection. Most studies are retrospective and subject to selection bias, potential immortal-time bias, and differences in the timing and choice of subsequent chemotherapy. Accordingly, the potential association between prior ICI exposure and increased chemotherapy response rates requires further investigation in prospective studies [42,43,44,45].

In clinical practice, the choice of chemotherapy regimen should be individualized according to prior treatment exposure, histologic subtype, performance status, comorbidities, and other relevant clinical factors [31,41].

### 3.2. Choice of Chemotherapy Regimens

Selection of chemotherapy after immunotherapy should be guided by histologic subtype, previous systemic therapy, performance status, comorbidities, and molecular characteristics. In patients who have not previously received platinum-based chemotherapy, platinum-doublet chemotherapy remains an appropriate option. Because most patients with advanced NSCLC who receive first-line ICI-based therapy have already been exposed to platinum-containing chemotherapy, subsequent treatment after ICI failure generally consists of non-platinum chemotherapy [31,41].

Among these options, docetaxel with or without ramucirumab (RAM/DOC) has been extensively studied and is an important option in the post-ICI setting. The phase III REVEL trial demonstrated that RAM/DOC improved OS and PFS compared with docetaxel alone in patients with advanced NSCLC [47]. Subsequent retrospective studies have suggested that this activity is maintained after prior PD-1/PD-L1 blockade [48,49]. In one study, the ORR was 60% and the median PFS was 5.6 months after nivolumab failure [48].

In the phase II SCORPION study, RAM/DOC administered after first-line platinum-based chemotherapy plus an ICI resulted in an ORR of 34.4% and a median PFS of 6.5 months [50]. In the multicenter phase II DRUN study, which evaluated RAM/DOC administered immediately after progression on an ICI-containing regimen, the ORR was 33.3%, the disease control rate (DCR) was 90.9%, and the median PFS and OS were 4.9 and 11.8 months, respectively [51]. Although the primary endpoint was not met, these prospective studies provide evidence that RAM/DOC retains antitumor activity after ICI exposure. However, no randomized trial has directly compared RAM/DOC with other chemotherapy regimens specifically in the post-ICI setting.

The potential activity of RAM/DOC after ICI exposure has been hypothesized to reflect interactions between VEGF pathway inhibition and the tumor immune microenvironment. VEGF signaling contributes to an immunosuppressive tumor microenvironment by impairing dendritic-cell function, promoting regulatory T cells and myeloid-derived suppressor cells, and inhibiting effective T-cell trafficking. VEGF pathway inhibition may therefore partially reverse these immunosuppressive effects and potentially complement residual immune activity after ICI treatment [52]. However, this biological rationale remains largely hypothetical in the post-ICI setting, and a causal relationship between prior ICI exposure and increased sensitivity to RAM/DOC has not been established.

Other cytotoxic chemotherapy options remain relevant after ICI failure. Pemetrexed is an established option for patients with nonsquamous NSCLC who have not previously received pemetrexed. In a randomized phase III trial of previously treated advanced NSCLC, pemetrexed demonstrated overall survival comparable to docetaxel (8.3 vs. 7.9 months), with substantially fewer grade 3–4 hematologic toxicities [53]. Nab-paclitaxel is another potential option; in the phase III J-AXEL trial, nab-paclitaxel was noninferior to docetaxel in overall survival and achieved a higher objective response rate, though its toxicity profile differed from that of docetaxel [54]. Gemcitabine also remains a potential subsequent-line option, particularly when other regimens are unsuitable [31,41].

At present, no chemotherapy regimen has demonstrated clear superiority specifically in the post-immunotherapy setting. Although RAM/DOC has been evaluated in prospective phase II studies in this setting, its superiority over other chemotherapy regimens has not been demonstrated in a randomized trial. Treatment selection should therefore be individualized according to previous therapies, histologic and molecular characteristics, comorbidities, expected toxicities, performance status, and patient preferences.

### 3.3. Limitations and Future Directions of Chemotherapy After ICI Failure

Despite encouraging clinical findings, several important limitations should be acknowledged: most available studies evaluating chemotherapy after ICI exposure are retrospective and include relatively small or heterogeneous patient populations; differences in prior ICI regimens, duration of treatment response, PD-L1 expression, molecular characteristics, and subsequent chemotherapy further complicate interpretation of the available evidence [42,43,44,45,48,49,50,51]; and prospective randomized studies directly comparing different post-ICI chemotherapy strategies remain lacking.

Predictive biomarkers for identifying patients most likely to benefit from chemotherapy after ICI therapy are also poorly defined. Dynamic biomarkers, including ctDNA, CTCs, and longitudinal immune profiling, may provide opportunities for more precise treatment selection, though their clinical utility remains to be established [21].

Beyond conventional cytotoxic chemotherapy, antibody–drug conjugates (ADCs) represent an emerging therapeutic approach for previously treated NSCLC. In the phase III TROPION-Lung01 trial, the TROP-2-directed ADC datopotamab deruxtecan significantly improved PFS compared with docetaxel (4.4 vs. 3.7 months, *p* = 0.004), though OS was not significantly improved in the overall study population (12.9 vs. 11.8 months, *p* = 0.53) [55]. Trastuzumab deruxtecan has also demonstrated clinical activity in patients with HER2-mutant NSCLC [56]. However, these findings should not be interpreted as establishing ADCs as a standard treatment strategy for all patients whose disease progresses after ICI-based therapy. Further studies are needed to clarify their efficacy in specific post-ICI populations, identify predictive biomarkers, and determine optimal treatment sequencing.

Combination strategies involving ICIs and other agents have also been investigated after progression on prior ICI-based treatment. In the randomized phase II Lung-MAP S1800A trial, pembrolizumab plus ramucirumab was associated with longer OS than standard-of-care therapy (14.5 vs. 11.6 months, *p* = 0.05), though PFS and ORR were similar between the treatment groups (PFS; 4.5 vs. 5.2 months, *p* = 0.25) [57]. However, the subsequent phase III PRAGMATICA-LUNG (S2302) trial did not demonstrate an OS benefit with pembrolizumab plus ramucirumab compared with standard-of-care therapy (10.1 vs. 10.0 months, *p* = 0.66) [58]. These findings highlight the importance of confirmatory randomized trials and caution against assuming that promising phase II findings will translate into improved survival in larger studies.

Prospective clinical trials are needed to define optimal treatment sequencing, identify biomarkers for treatment selection, and evaluate novel combination strategies after ICI failure. As the use of perioperative immunotherapy continues to expand, the management of patients who develop recurrence after adjuvant or neoadjuvant ICI therapy will become an increasingly important clinical and research question. Determining whether prior immunotherapy influences the efficacy of subsequent chemotherapy in this setting may further refine treatment strategies for recurrent disease.

## 4. Local Therapy After Immunotherapy Failure

### 4.1. Rationale for Local Therapy

The clinical presentation of disease progression following ICI therapy is highly heterogeneous. While some patients develop rapid systemic progression requiring an immediate change in systemic therapy, others experience progression confined to a limited number of lesions despite continued disease control at other sites. This pattern, commonly referred to as OPD, has important therapeutic implications [33,34].

In selected patients with OPD, local treatment may provide control of progressing lesions while allowing for the continuation of systemic therapy that remains effective at other disease sites [11,25].

This treatment concept is similar to the management of oligoprogression in oncogene-driven NSCLC treated with tyrosine kinase inhibitors (TKIs), in which local ablative therapy can permit the continuation of an otherwise effective systemic treatment in selected patients [59,60]. Local treatment may therefore prolong the duration of disease control while delaying the initiation of subsequent systemic therapy.

Accumulating clinical evidence suggests that selected patients with OPD may benefit from local ablative therapy while continuing effective systemic treatment [33,34]. However, the optimal selection of candidates, timing of local treatment, and role of continued ICI therapy remain incompletely defined.

### 4.2. Radiotherapy for Oligoprogressive Disease

Among local treatment modalities, SBRT is an important approach to treating OPD because it can provide effective local control with limited treatment interruption and morbidity. In the context of ICI therapy, SBRT has also attracted interest because of its potential immunomodulatory effects.

Beyond direct tumor cell killing, radiotherapy can promote immunogenic cell death, release tumor-associated antigens and damage-associated molecular patterns, activate dendritic cells, and enhance tumor-specific T-cell responses. These effects provide a biological rationale for combining radiotherapy with immune checkpoint blockade [61,62,63]. However, radiotherapy may also damage local immune structures, including tertiary lymphoid structures (TLSs), and its immunomodulatory effects may vary depending on treatment parameters such as dose and fractionation [64].

The abscopal effect, defined as regression of nonirradiated tumor lesions following localized radiotherapy, represents the most striking example of the potential interaction between radiotherapy and systemic antitumor immunity. Although the biological basis of the abscopal effect is increasingly understood, clinically meaningful abscopal responses remain uncommon and unpredictable, and reliable predictive biomarkers have not been established [65]. Furthermore, a randomized phase II trial by Schoenfeld et al. found that adding low-dose or hypofractionated radiotherapy to durvalumab plus tremelimumab did not improve response rates in patients with PD-(L)1-refractory NSCLC [66]. These findings indicate that the clinical efficacy of radiation-mediated immune enhancement remains unestablished.

Nevertheless, clinical studies have demonstrated the benefit of local radiotherapy in oligoprogressive NSCLC. In a multicenter retrospective study of 61 patients with oligoprogressive NSCLC receiving first-line immunotherapy, local ablative radiotherapy achieved a DCR of 89% [33]. More importantly, the phase II CURB trial prospectively evaluated SBRT in patients with oligoprogressive metastatic NSCLC and demonstrated significantly prolonged PFS with SBRT plus standard systemic therapy compared with standard systemic therapy alone in the NSCLC subgroup, with a median PFS of 10.0 versus 2.2 months (HR, 0.41; 95% CI, 0.22–0.75) [34].

The clinical benefit of radiotherapy in oligoprogressive NSCLC may reflect not only local control of progressing lesions but also potential immunomodulatory effects, although the specific contribution of the latter remains unclear. The evidence base remains heterogeneous with respect to the definition of OPD, number and location of progressive lesions, timing of radiotherapy, systemic treatment, and whether immunotherapy was continued beyond progression. Thus, SBRT may be considered primarily for carefully selected patients in whom systemic disease remains controlled and all progressing lesions can be safely treated.

### 4.3. Salvage Surgery

Salvage surgery represents another local treatment option for highly selected patients with localized residual or recurrent disease following immunotherapy. Surgical resection may be considered when disease remains anatomically resectable and systemic disease is otherwise controlled. Potential candidates may include patients with persistent localized disease after a major response to immunotherapy, isolated local recurrence, or OPD confined to technically resectable lesions [20,67,68,69,70].

In some cases, surgical resection may also provide definitive pathological assessment when radiographic findings are insufficient to distinguish residual viable tumor from treatment-related changes. An additional potential advantage is the availability of surgical specimens for pathological and molecular assessment, which may provide information regarding treatment response and mechanisms of resistance and thereby facilitate subsequent treatment decisions and translational research.

Several retrospective studies have demonstrated the feasibility of pulmonary resection after ICI therapy, with acceptable perioperative outcomes in appropriately selected patients [20,67,68,69,70]. However, previous immunotherapy may be associated with substantial inflammatory and fibrotic changes around the tumor, hilum, and lymph nodes, potentially increasing technical difficulty during dissection. Accordingly, salvage surgery should be performed in experienced centers after careful multidisciplinary assessment of resectability and expected perioperative risk.

Recent multicenter data further support the feasibility of salvage surgery after ICI therapy. In a multicentric retrospective study of 31 patients with stage III–IVA NSCLC who were initially considered unresectable and subsequently underwent radical-intent surgery after ICI-based treatment, postoperative complications occurred in 25% of patients, while complete and major pathologic responses were observed in 48% and 16%, respectively. The 2- and 3-year survival rates were both 88% [69]. These findings support the feasibility of salvage surgery in selected responders, though the retrospective design and highly selected patient population preclude conclusions regarding its comparative oncologic benefit. A more recent 14-center Japanese retrospective study of 32 patients similarly demonstrated the feasibility of salvage surgery after immunotherapy, though the retrospective design and heterogeneous indications for surgery limit interpretation of its oncologic benefit [70].

Because the available evidence consists predominantly of retrospective studies from selected institutions, the optimal indications, timing, and oncologic benefit of salvage surgery after ICI therapy remain uncertain.

### 4.4. Other Local Ablative Modalities

Other local ablative modalities, including radiofrequency ablation, microwave ablation, and cryoablation, may provide local control for selected lesions, particularly when surgery or SBRT is not feasible. Thermal ablation is an established local treatment option for selected patients with lung cancer, with the choice of modality depending on tumor size, location, and technical considerations [71].

However, clinical evidence specifically evaluating these approaches for oligoprogressive NSCLC after ICI therapy remains limited. Although thermal ablation may have immunomodulatory effects and could potentially complement immune checkpoint blockade, the clinical efficacy of such strategies remains uncertain [72].

Consequently, these modalities generally have more selective indications than SBRT or surgery in the management of oligoprogressive NSCLC after immunotherapy. Their use should be individualized according to lesion location, technical feasibility, prior treatment, anticipated toxicity, and institutional expertise.

### 4.5. Multidisciplinary Management and Future Perspectives

Optimal management of OPD requires multidisciplinary assessment involving medical oncologists, radiation oncologists, and thoracic surgeons. Treatment selection should consider the number and distribution of progressive lesions, previous response and duration of benefit from ICI therapy, performance status, technical feasibility of local treatment, and the availability of effective systemic treatment options [33,34].

Several important questions remain unresolved. These include the optimal definition of OPD, the number and location of lesions suitable for local treatment, the preferred local treatment modality, and whether ICI therapy should be continued after local treatment [33,34]. Prospective studies are needed to better define which patients may benefit from local therapy while maintaining effective systemic treatment and which patients should instead undergo a change in systemic therapy.

Dynamic biomarkers, including ctDNA, CTCs, and longitudinal immune profiling, may eventually facilitate individualized treatment selection, though their clinical utility in this setting remains to be established [21].

As perioperative immunotherapy becomes increasingly incorporated into the treatment of resectable NSCLC, recurrence after neoadjuvant or adjuvant ICI-based therapy will represent an increasingly important clinical scenario [7,8,9,10]. Future studies should therefore investigate whether local treatment can provide durable disease control in selected patients with limited recurrence after perioperative immunotherapy.

## 5. ICI Rechallenge

### 5.1. General Principles and Clinical Context of ICI Rechallenge

The increasing use of ICIs in advanced NSCLC has created several clinical scenarios in which re-exposure to immunotherapy may be considered. However, the term “ICI rechallenge” encompasses biologically and clinically distinct situations, and these should not be considered interchangeable. In particular, rechallenge after disease progression during active ICI therapy differs from retreatment after planned treatment completion or rechallenge after treatment discontinuation because of irAEs.

The potential benefit of ICI re-exposure appears to depend substantially on the reason for discontinuation of the initial ICI. Retrospective studies and meta-analyses have generally reported modest activity of ICI rechallenge after disease progression, whereas greater activity has been reported in selected patients whose initial ICI was discontinued for reasons other than progressive disease [73,74,75,76].

The available evidence is predominantly derived from retrospective studies, single-arm prospective trials, and selected populations, making patient selection a critical component of ICI rechallenge. Factors that may influence the potential benefit of re-exposure include the depth and duration of the initial response, the reason for treatment discontinuation, the interval without ICI therapy, the pattern of subsequent disease progression, the presence of actionable genomic alterations, and the availability of alternative systemic or local treatment options. Importantly, a previous response to ICI therapy alone does not necessarily predict benefit from rechallenge after acquired resistance [74].

Accordingly, ICI re-exposure should be regarded not as a uniform treatment strategy but as a context-dependent approach. The following sections separately address rechallenge after disease progression and rechallenge after irAEs, while distinguishing these situations from retreatment after planned treatment completion and continuation of ICI therapy with local treatment for OPD.

### 5.2. Rechallenge After Disease Progression

The ESMO Clinical Practice Guideline states that anti-PD-(L)1 rechallenge may be considered in selected patients who previously derived substantial clinical benefit and discontinued ICI therapy for reasons other than disease progression, whereas rechallenge is generally not recommended when treatment was discontinued because of progressive disease [31].

Prospective data support the limited activity of ICI rechallenge after acquired resistance. In the phase II WJOG9616L study, patients with advanced NSCLC who had previously achieved CR, PR, or SD for at least 6 months and subsequently experienced disease progression after an ICI-free interval of at least 60 days received nivolumab retreatment. The ORR was 8.5%, and the median PFS was 2.6 months. The ICI-free interval was associated with subsequent PFS, whereas prior response and a history of irAEs were not [74]. These findings suggest that a previous response to ICI therapy alone does not reliably predict benefit from rechallenge once acquired resistance has emerged. Meta-analyses have similarly demonstrated substantially lower response rates with ICI rechallenge than with initial ICI treatment, though outcomes appear to differ according to the reason for initial ICI discontinuation [73].

Retreatment after planned completion of ICI therapy represents a distinct clinical setting. In KEYNOTE-010, patients who completed 2 years of pembrolizumab could receive a second course after subsequent disease progression; among the 14 patients who received retreatment, six achieved a partial response and five had stable disease [75]. However, these findings should not be directly extrapolated to patients who develop progressive disease during active ICI therapy.

Another distinct scenario is OPD, in which progression is limited to a small number of lesions while other disease sites remain controlled by systemic therapy. In this setting, continuation of ICI therapy with local ablative treatment may be considered rather than immediate discontinuation of immunotherapy. Retrospective studies and the randomized phase II CURB trial have provided evidence supporting local ablative treatment in selected patients with oligoprogressive NSCLC [33,34]. Importantly, this strategy should be distinguished from ICI rechallenge because immunotherapy is continued rather than reintroduced after systemic progression.

Finally, recurrence after completion of perioperative immunotherapy represents an emerging clinical scenario. As perioperative ICI-based regimens become increasingly incorporated into the treatment of resectable NSCLC, recurrence after prior exposure to perioperative immunotherapy will become increasingly relevant [7,8,9,10]. However, the optimal systemic treatment for recurrence after perioperative ICI remains undefined, and prospective evidence supporting ICI rechallenge in this setting is currently limited.

### 5.3. Rechallenge After Immune-Related Adverse Events

Rechallenge after discontinuation of ICI therapy because of irAEs represents a distinct clinical scenario in which the potential for renewed antitumor activity must be balanced against the risk of recurrent immune toxicity. The decision to resume ICI therapy should take into account the type and severity of the initial irAE, the degree and duration of tumor response, the time required for toxicity resolution, the availability of alternative treatment options, and the patient’s performance status [39,40,76,77].

Available retrospective studies and meta-analyses indicate that ICI rechallenge is feasible in selected patients after the resolution of irAEs, but recurrent toxicity remains a clinically relevant risk. A recent meta-analysis of patients with NSCLC reported that ICI resumption after irAEs was associated with improved response and survival compared with non-resumption; however, irAEs occurred in approximately 45% of patients after treatment resumption [39]. In a large pharmacovigilance analysis, recurrence of the same irAE occurred in 28.8% of patients after ICI rechallenge [77]. These estimates should be interpreted in light of differences in study populations, patient selection, and definitions of recurrent toxicity. Rechallenge may also lead to a different irAE, and colitis, hepatitis, and pneumonitis have been associated with relatively high recurrence risks in some studies [39,49,76,77].

According to the ASCO guideline, ICI therapy may generally be resumed after most grade 2 toxicities once symptoms and laboratory abnormalities have resolved to grade 1 or less. Rechallenge may also be considered in selected patients with grade 3 toxicity after appropriate recovery, though organ-specific recommendations and clinical judgment are essential. In contrast, grade 4 toxicities generally warrant permanent discontinuation of ICI therapy, with the exception of endocrine toxicities that are adequately controlled with hormone replacement [78]. The risk associated with rechallenge varies according to the affected organ. Endocrine toxicities, including hypothyroidism and adrenal insufficiency, generally do not preclude subsequent ICI therapy when adequately controlled with hormone replacement. In contrast, severe pneumonitis, myocarditis, and serious neurologic toxicities warrant particular caution and may require permanent discontinuation. The ASCO guideline recommends permanent discontinuation for grade 3 or higher neurologic toxicities, while myocarditis and severe pneumonitis also warrant permanent discontinuation or strong consideration of permanent discontinuation depending on the clinical context [78].

Accordingly, rechallenge after an irAE should be regarded as an individualized risk–benefit decision rather than a routine treatment strategy. Multidisciplinary consultation with organ-specific specialists is particularly important when rechallenge is being considered after severe or potentially life-threatening toxicity.

### 5.4. Strategies to Improve the Efficacy of Rechallenge

Because response rates to single-agent ICI rechallenge remain modest, combination strategies aimed at overcoming mechanisms of resistance are being actively investigated.

One approach involves reintroducing ICI therapy in combination with cytotoxic chemotherapy: chemotherapy may enhance tumor antigenicity through immunogenic cell death and modulate the tumor microenvironment, providing a potential biological rationale for combining chemotherapy with immune checkpoint blockade [46]. However, clinical evidence specifically supporting chemotherapy plus ICI rechallenge after acquired resistance remains limited, and the optimal chemotherapy regimen and patient population have not been established.

Another strategy involves combining ICIs with antiangiogenic agents. Antiangiogenic therapy may enhance antitumor immunity through vascular normalization, improvement of immune-cell infiltration, and modulation of immunosuppressive myeloid populations [52]. This provides a biological rationale for combining antiangiogenic therapy with ICI rechallenge, though clinical evidence specifically evaluating this strategy after acquired resistance remains limited.

Beyond conventional combination approaches, next-generation immunotherapies targeting alternative immune checkpoints, including LAG-3, TIGIT, TIM-3, and VISTA, are being evaluated as potential strategies to overcome primary or acquired resistance to PD-1/PD-L1 blockade. These approaches remain investigational, and their clinical benefit in patients specifically undergoing ICI rechallenge has not yet been established [79].

Personalized rechallenge strategies incorporating molecular profiling, tumor immune characteristics, and dynamic biomarkers may further improve patient selection. Genomic and immunophenotypic alterations associated with acquired resistance, together with longitudinal changes in the tumor microenvironment, may ultimately help identify patients who are more likely to benefit from specific combination or sequential treatment strategies [21,32,79].

### 5.5. Limitations and Future Directions of ICI Rechallenge

Despite increasing clinical experience, several important questions regarding ICI rechallenge remain unanswered. Available evidence has been limited by heterogeneous patient populations, inconsistent definitions of rechallenge and resistance, and variable intervals between treatment courses. Recent systematic reviews and meta-analyses have provided more robust evidence, but the overall clinical benefit of PD-(L)1-based rechallenge remains modest [73,80]. In particular, no clear benefit has been demonstrated in patients with primary resistance, whereas patients with features of acquired resistance may derive greater benefit from rechallenge strategies. However, these findings remain exploratory because resistance phenotypes have been defined inconsistently across studies [12,73,80].

Future studies should focus on identifying biomarkers capable of distinguishing patients who are likely to benefit from rechallenge from those who require alternative therapeutic strategies. Dynamic biomarkers, including ctDNA, CTCs, and longitudinal assessment of tumor and immune characteristics, may provide valuable information regarding treatment response, clonal evolution, and mechanisms of resistance [21,32,81,82,83,84,85,86]. Further integration of molecular profiling with immune-related biomarkers and serial liquid biopsy approaches may facilitate more precise patient selection and treatment adaptation.

Ultimately, personalized rechallenge strategies integrating clinical characteristics, resistance phenotypes, molecular alterations, and dynamic biomarkers may become an important component of precision immuno-oncology.

The principal treatment strategies after immunotherapy failure and their supporting evidence are summarized in Table 1.

## 6. Biomarkers and Future Perspectives

### 6.1. Why Are Biomarkers Needed After Immunotherapy Failure?

Although ICIs have transformed the treatment landscape of lung cancer, disease progression after immunotherapy represents a biologically heterogeneous condition rather than a single clinical entity. Patients with primary resistance, acquired resistance, oligoprogression, or recurrence following treatment discontinuation may exhibit distinct tumor–immune interactions and therefore require different therapeutic strategies. Consequently, biomarkers capable of characterizing resistance mechanisms and informing subsequent treatment selection represent an important unmet need in thoracic oncology [21,25,26,32].

Current treatment selection after ICI failure relies largely on clinical factors, including prior treatment response, duration of disease control, performance status, and the pattern of progression. However, these parameters provide limited insight into the underlying biological mechanisms of resistance. Integrating molecular and immune biomarkers with clinical resistance phenotypes may therefore facilitate a transition from empiric treatment sequencing toward biomarker-guided precision immuno-oncology [21,32].

An important challenge is that the biological state of a tumor may evolve during immunotherapy: tumor cells can undergo clonal selection and acquire genomic or immunophenotypic changes under immune pressure, while the tumor immune microenvironment may also undergo dynamic remodeling [32]. Consequently, biomarkers obtained before ICI treatment may not accurately reflect the biological state of tumors at the time of progression, underscoring the potential value of longitudinal biomarker assessment [32,81].

### 6.2. Tissue Biomarkers

Tissue-based biomarkers remain central to the assessment of tumor biology and immunotherapy sensitivity in NSCLC. Among these, PD-L1 expression is the most widely used biomarker for guiding immunotherapy selection in advanced NSCLC. However, its predictive value after immunotherapy failure is limited by the dynamic nature of PD-L1 expression, which may change following exposure to ICIs, chemotherapy, or radiation therapy. Furthermore, substantial intratumoral and intertumoral heterogeneity may reduce the reliability of single-site biopsy specimens [21,32].

Tumor mutational burden (TMB) has also been extensively investigated as a predictor of immunotherapy efficacy. Although a high TMB may increase neoantigen generation and enhance antitumor immune responses, prospective studies have produced inconsistent results, and TMB has not been established as a routine biomarker for treatment selection after ICI progression [21].

Genomic alterations associated with immune resistance may provide additional information regarding tumor biology after ICI failure. In particular, alterations in STK11 and KEAP1/NFE2L2 have been associated with reduced sensitivity to PD-1/PD-L1 blockade, particularly in KRAS-mutant NSCLC [27,28]. Other alterations, including abnormalities involving antigen-presentation pathways and interferon-γ signaling, may contribute to acquired resistance through impaired tumor recognition and immune escape [14,32]. However, the clinical utility of these alterations as predictive biomarkers for treatment selection after ICI failure remains to be established.

### 6.3. Liquid Biopsy

Liquid biopsy has emerged as a promising approach for monitoring treatment response and resistance during immunotherapy. Compared with conventional tissue biopsy, circulating biomarkers can be repeatedly assessed using minimally invasive procedures and may provide complementary information on the spatial and temporal heterogeneity of advanced lung cancer [81].

#### 6.3.1. *Circulating Tumor DNA*

Among liquid biopsy biomarkers, ctDNA has accumulated substantial clinical evidence. Multiple studies have demonstrated that early clearance or reduction in ctDNA during systemic therapy, including immune checkpoint blockade, is associated with prolonged PFS and OS. Conversely, persistent or increasing ctDNA levels may precede radiographic progression, providing an opportunity for earlier detection of emerging resistance. A recent systematic review and meta-analysis of 32 studies involving 3047 patients with advanced NSCLC confirmed that ctDNA reduction or clearance was associated with improved PFS and OS, including in patients receiving immune checkpoint blockade [82].

However, the current evidence primarily supports the prognostic and pharmacodynamic value of ctDNA kinetics rather than its established use as a predictive biomarker for selecting subsequent therapy after ICI failure. Prospective studies are therefore needed to determine whether ctDNA-guided treatment adaptation can improve clinical outcomes.

#### 6.3.2. *Circulating Tumor Cells*

CTCs provide complementary biological information because intact tumor cells can be characterized phenotypically and molecularly in ways that cannot be achieved using ctDNA alone. Beyond simple enumeration, assessment of epithelial–mesenchymal transition (EMT), PD-L1 expression, genomic alterations, and transcriptomic profiles may provide insights into tumor evolution and mechanisms of immune escape [81,83,84].

Emerging technologies enable increasingly comprehensive characterization of CTCs at the single-cell level, including integrated genomic, transcriptomic, and protein analyses. These approaches may facilitate longitudinal assessment of tumor evolution during immunotherapy, though their clinical utility remains investigational [83].

Combined analysis of ctDNA and CTCs may provide complementary information regarding tumor burden, clonal evolution, and phenotypic changes during treatment. Such integrated liquid biopsy approaches are therefore promising for individualized treatment monitoring, but their ability to guide treatment selection after immunotherapy failure remains to be validated prospectively [81].

### 6.4. Emerging Biomarkers

Rapid advances in translational oncology have expanded the spectrum of candidate biomarkers beyond conventional genomic profiling. Single-cell technologies enable high-resolution characterization of the tumor immune microenvironment (TIME), revealing substantial heterogeneity among immune-cell populations and identifying cellular states that may be relevant to response and resistance to immune checkpoint blockade [85]. In parallel, spatial transcriptomics and multiplex imaging approaches enable characterization of the spatial organization and interactions among tumor cells, cytotoxic T lymphocytes, regulatory T cells, macrophages, and stromal components. In a recent study of 234 patients with advanced NSCLC treated with PD-1-based immunotherapy, spatial proteomics and transcriptomics identified distinct cellular and cell-to-gene signatures associated with treatment response and resistance, highlighting the potential of spatial multi-omics for biomarker development [86].

Additional investigational biomarkers include T-cell receptor (TCR) repertoire characteristics, circulating immune-cell subsets, cytokine signatures, extracellular vesicle- or exosomal RNA, gut microbiome composition, and radiomic features derived from computed tomography images. These approaches may provide complementary information on systemic immune status, tumor–immune interactions, and dynamic treatment response. Emerging evidence suggests that gut microbiome composition is associated with clinical outcomes following ICI therapy in NSCLC, though the specific microbial signatures and their clinical applicability remain incompletely defined [87]. Radiomic approaches may also provide noninvasive information on tumor phenotype and treatment response; however, methodological heterogeneity and the lack of prospective external validation currently limit their clinical implementation [88].

Although these approaches remain largely exploratory, their integration with tissue-based biomarkers, liquid biopsy, and other multi-omics platforms may provide a more comprehensive assessment of tumor–immune interactions and dynamic treatment response. Ultimately, multimodal biomarker strategies may facilitate more precise characterization of resistance mechanisms and support individualized treatment selection after immunotherapy failure, but their clinical utility requires prospective validation [85,86,87,88].

### 6.5. Future Directions

Future management of patients experiencing immunotherapy failure will likely require integrated, biomarker-driven treatment strategies rather than empirical therapeutic sequencing. A precision medicine framework may incorporate multiple complementary dimensions, including the clinical resistance phenotype with dynamic liquid biopsy, tissue-based molecular and immune profiling, and spatial and single-cell characterization of the tumor microenvironment. Such integration may enable more precise characterization of tumor evolution and resistance mechanisms over the course of treatment [81,83,84,85,86,87,88].

An important future goal is to translate these emerging biomarkers from prognostic or exploratory tools into clinically actionable biomarkers. Prospective clinical trials should therefore move beyond retrospective biomarker discovery and evaluate whether biomarker-guided treatment allocation and longitudinal treatment adaptation can improve outcomes. Such studies will be essential to determine whether personalized, biomarker-driven treatment strategies can ultimately be incorporated into routine clinical practice.

## 7. Conclusions and Clinical Perspectives

The widespread integration of ICIs into the treatment paradigm of lung cancer has fundamentally transformed the management of patients across metastatic, locally advanced, and resectable disease settings. Nevertheless, disease progression during or after immunotherapy and treatment discontinuation because of irAEs remains an important unresolved challenge in contemporary thoracic oncology [1,2,3,4,5,6,7,8,9,10,12].

A central concept emphasized throughout this review is that immunotherapy failure should not be regarded as a single clinical entity. Primary resistance, acquired resistance, oligoprogression, hyperprogressive disease, and progression following treatment discontinuation represent biologically and clinically distinct scenarios that may require different therapeutic approaches. The accurate characterization of these resistance phenotypes therefore provides an important foundation for subsequent treatment selection [12,17,18,19].

Cytotoxic chemotherapy remains an important systemic treatment option after immunotherapy failure, particularly for patients without actionable molecular alterations. Among the available regimens, docetaxel plus ramucirumab has demonstrated clinical activity in the post-ICI setting, though its superiority over other chemotherapy strategies has not been established in randomized trials [48,49,50,51]. Local ablative therapy, including radiotherapy and, in highly selected patients, salvage surgery, may provide additional treatment options for oligoprogressive or otherwise locally controllable disease [33,34,63,65,67,68]. ICI rechallenge may be considered in selected patients, particularly those who have previously derived prolonged clinical benefit and discontinued immunotherapy for reasons other than progressive disease, whereas its role after acquired resistance or unequivocal progression during active ICI therapy remains limited and incompletely defined [73,74,75,76,77].

Looking forward, management after immunotherapy failure is expected to evolve from empirical treatment sequencing toward precision immuno-oncology. The integration of clinical resistance phenotypes with tissue biomarkers, ctDNA, CTCs, spatial and single-cell immune profiling, and other multi-omics approaches may improve the characterization of tumor evolution and facilitate individualized treatment selection [32,81,83,84,85,86]. However, most currently available biomarkers remain under investigation, and prospective validation is required before they can be routinely incorporated into clinical decision-making.

In parallel, next-generation immunotherapeutic approaches targeting alternative immune-regulatory pathways may expand the therapeutic options available to patients whose tumors progress despite conventional PD-1/PD-L1 blockade [79]. However, their clinical efficacy, specifically in patients with established ICI resistance, remains to be determined [79].

Ultimately, successful management after ICI failure will require the multidisciplinary integration of medical oncology, thoracic surgery, radiation oncology, pathology, radiology, and translational research. Future clinical trials should incorporate standardized definitions of resistance, longitudinal molecular monitoring, and biomarker-guided treatment allocation to determine which patients are most likely to benefit from chemotherapy, local therapy, ICI re-exposure, targeted therapy, or emerging immunotherapeutic strategies. Such an approach may provide the foundation for evidence-based, individualized treatment algorithms and further advance precision immuno-oncology in lung cancer.

## Figures and Tables

**Table 1:** Treatment strategies after immunotherapy failure in NSCLC.

Treatment Strategy	Clinical Setting	Summary of Evidence and Clinical Role	Key Limitations	Representative Evidence
A. Systemic chemotherapy				
Cytotoxic chemotherapy	Disease progression during or after ICI therapy	Remains an important subsequent-line treatment option. Activity may be retained after prior ICI exposure.	Post-ICI evidence is mainly retrospective or non-randomized.	Retrospective studies [42,43,44], WJOG10217L [45]
Docetaxel + ramucirumab	Disease progression after ICI-based therapy	Established subsequent-line option with clinical activity after ICI exposure.	No randomized evidence specific to post-ICI failure.	REVEL [47], retrospective studies [48,49], SCORPION [50], DRUN [51]
Pemetrexed	Previously treated nonsquamous NSCLC	Established option for eligible patients without prior pemetrexed exposure.	Evidence is not specific to the post-ICI setting.	Phase III comparative study [53]
Nab-paclitaxel	Previously treated NSCLC	Alternative cytotoxic option with noninferior OS to docetaxel in J-AXEL.	Not specifically evaluated after ICI failure.	J-AXEL [54]
Gemcitabine	Previously treated NSCLC	Remains a subsequent-line cytotoxic option, particularly when other regimens are unsuitable.	Evidence is not specific to the post-ICI setting.	ESMO and ASCO guidelines [31,41]
B. Local therapy				
SBRT or other local ablative therapy	Oligoprogressive disease with otherwise controlled systemic disease	May provide local control while allowing continuation of effective systemic therapy.	Patient selection and prospective evidence remain limited.	Retrospective study [33], CURB [34]
Salvage surgery	Selected localized residual or recurrent disease after ICI-based therapy	May provide definitive local treatment in carefully selected patients.	Small selected cohorts, with long-term benefit remaining uncertain.	Retrospective studies [20,67,68,69,70]
Other ablative modalities	Limited lesions amenable to local treatment	May provide local control when surgery or SBRT is unsuitable.	Limited post-ICI evidence, with varying technical feasibility.	Reviews [71,72]
C. ICI rechallenge				
ICI rechallenge after disease progression	Progression during active ICI therapy	Generally limited activity and not established as routine treatment after unequivocal progression.	Limited prospective evidence with no validated selection criteria.	Meta-analysis [73], WJOG9616L [74]
ICI re-exposure after irAEs	Prior clinical benefit with resolution or adequate control of irAEs	May be considered selectively after careful assessment of prior toxicity and expected benefit.	Risk of recurrent or new irAEs.	Meta-analysis [39], retrospective study [76], pharmacovigilance study [77], ASCO guideline [78]
Retreatment after planned treatment completion	Disease recurrence after completion of a planned ICI course	May have activity in selected patients after a treatment-free interval.	Limited evidence, with optimal selection remaining uncertain.	Meta-analysis [73], KEYNOTE-010 [75]

ADCs, Antibody-drug conjugates; ICI, immune checkpoint inhibitor; irAEs, immune-related adverse events; NSCLC, non-small cell lung cancer; OS, overall survival; SBRT, stereotactic body radiotherapy.

## Data Availability

No new data were created or analyzed in this study. Data sharing is not applicable to this article.
